# Pulsed Versus Conventional Radiofrequency Stimulation in Cervical Facet-Mediated Neck Pain: A Single-Centre Retrospective Cohort Study Outcomes

**DOI:** 10.3390/healthcare14060819

**Published:** 2026-03-23

**Authors:** Derya Bayram, Çağatay Küçükbingöz

**Affiliations:** Department of Pain Medicine, Adana City Training and Research Hospital, Ministry of Health, 01370 Adana, Turkey; ckbingoz.md@gmail.com

**Keywords:** radiofrequency ablation, zygapophyseal joint, neck, pain

## Abstract

**Background/Objectives**: Cervical facet-mediated pain is a significant underlying factor of persistent neck pain (CNP). Pulsed radiofrequency (PRF) or conventional radiofrequency (CRF) has been introduced as a treatment alternative. However, comparative clinical data remain limited. **Methods**: This single-center retrospective cohort study analyzed patients with cervical facet-mediated pain who underwent PRF (n = 40) or CRF (n = 44) between January 2023 and December 2024. The success of the procedure was assessed using the Numeric Rating Scale (NRS) and the Neck Disability Index (NDI) before the procedures and at 1, 3, 6, and 12 months following the injections. Patients’ feedback was evaluated using the Global Perceived Effect (GPE) scale. **Results**: For both groups, a substantial decrease in the mean pain and disability severity was recorded between the initial measurement and the first, third, and sixth months of follow-up, but the outcomes were significant only in the CRF group at the 12th month. The groups did not show a substantial difference in terms of pain relief, disability improvement, medication use, or patient satisfaction at one and three months (*p* > 0.05), but at six and 12 months, patients treated with CRF showed significantly greater outcomes (*p* < 0.001). No notable difference in complication rates was found between the PRF (10%) and CRF (16%) groups (*p* = 0.53). **Conclusions**: Both pulsed and conventional radiofrequency ablation effectively reduced pain and improved function in the early-midterm follow-up. However, CRF provided more sustained relief and greater patient-reported success at 6 and 12 months, without an increase in complication rates, suggesting that CRF may offer a more durable long-term treatment option.

## 1. Introduction

Chronic neck pain (CNP), which continues for periods of greater than seven to 12 weeks, following non-surgical methods, is a common clinical problem with a lifetime prevalence reported between 54% and 80% [1]. Among the various etiologies, cervical facet joints are responsible for 36% to 67% of cases of long-term neck pain, even after trauma or surgery [2]. Facet-mediated pain significantly impacts the ability to function, quality of life, and work productivity [3].

The innervation of individual facet joints is supplied by the medial branch of the cervical dorsal ramus of the spinal nerve, both superiorly and inferiorly. According to the findings of anatomical studies, the trajectory of the C3–6 medial branches is positioned at the central aspect of the articular pillar. Pain attributed to the cervical zygapophyseal joints is classified as a facetogenic pain referral distribution, the nature of which depends on the level of the cervical zygapophyseal articulation [4].

Diverse therapeutic approaches, including oral medication, physical therapy, and facet joint corticosteroid injection, have been utilised for the control of facet-mediated pain [5,6]. However, these modalities frequently offer only transient relief, thus limiting their long-term utility. Interventional options for facet joint-related pain generally include intra-articular steroid injections, diagnostic or therapeutic medial branch blocks (MBBs), and radiofrequency neurotomy targeting the medial branch nerves using pulsed, conventional, or cooled radiofrequency modalities [7]. These blocks may provide clinical benefit by inhibiting ectopic neural discharges, reducing neurogenic inflammation, and modulating the sympathetic reflex arc [8]. By selectively lesioning the medial branch nerves, radiofrequency neurotomy blocks pain transmission from the affected facet joint to the spinal cord. Consequently, pain relief is restricted to the denervated joint and does not extend to other cervical pain generators [9].

Conventional radiofrequency (CRF) and pulsed radiofrequency (PRF) stimulation of the cervical medial branches (CMBs) has also been employed in clinical practice for long-lasting pain control and has become a widely accepted minimally invasive technique [10,11]. CRF produces analgesia through the generation of heat, leading to thermocoagulation and the destruction of neural tissue. It may also be associated with adverse effects such as the development of neuritis, dysesthesia, numbness, and transient motor weakness. Conversely, PRF delivers brief electrical current pulses at reduced temperatures, producing neuromodulatory effects without significant neural destruction. Therefore, PRF has been proposed as a safer alternative with fewer complications [12]. Furthermore, PRF interacts with diverse pathways involved in pain signalling, inflammatory processes, and synaptic plasticity [13].

Recent studies indicate that targeting the medial branch nerves with pulsed radiofrequency can lead to clinically meaningful improvements in pain and function [14,15]. In their study, Yen et al., in a retrospective study including 204 patients evaluating the effectiveness of PRF for cervical facet joint pain, reported a favorable clinical outcome in 68.1% of cases [16]. In a study by Chang et al., NRS scores in 21 patients were significantly lower at 1 and 3 months compared with baseline values. Notably, clinically meaningful pain relief persisted in 52.4% of patients at the 3-month follow-up [15].

Despite its increasing clinical use, high-quality comparative data directly evaluating the long-term effectiveness of PRF versus CRF in cervical facet-mediated pain remain limited. Therefore, the present study was designed to address this gap by directly comparing PRF and CRF applied to the CMBs in a well-defined CNP population. Unlike prior reports, this trial evaluates not only pain intensity but also functional impairment and patient satisfaction, thereby providing a more comprehensive assessment of clinical outcomes. By offering comparative long-term data, this study aims to clarify the relative effectiveness of these two commonly used interventions and contribute to the literature in the management of cervical facet-mediated pain.

## 2. Materials and Methods

### 2.1. Study Design and Ethical Approval

This retrospective cohort study was conducted at the Department of Pain Medicine at Adana City Training and Research Hospital. Ethical approval for the study was granted by the Institutional Review Board of Adana City Training and Research Hospital (Approval No: 920, Date: 18 December 2025). The electronic case notes of 95 individuals who received CMBs pulsed or conventional RFA between January 2023 and December 2024 were reviewed. Owing to the retrospective study design, written informed consent was not required. The study was conducted in accordance with the principles of the Declaration of Helsinki and was reported following the STROBE guidelines.

### 2.2. Inclusion and Exclusion Criteria

Chronic neck pain (CNP) was described as pain that persisted for over three months. Patients were also required to have axial cervical pain in the absence of radicular features, demonstrate ≥80% short-term pain reduction after diagnostic CMBs block (0.5 mL of 1% lidocaine per level), and report an NRS score of at least 4 before intervention. Patients were excluded if they presented with radicular neck pain, advanced cervical disc disease, a history of cancer, uncontrolled psychiatric disorders, progressive neurological deficits, an allergy to radio-opaque contrast medium or local anesthetic, or incomplete medical records. All patients who met the inclusion criteria during the study period were consecutively enrolled.

There were no limitations or alterations to the initial home pharmaceutical treatments. The RFA procedures were carried out at the levels corresponding to those confirmed by the diagnostic MBBs. Patients were considered for diagnostic procedures according to their presenting symptoms and the distribution of discomfort, the presence of paravertebral sensitivity on the impacted levels, the reproducibility of pain with deep pressure, and imaging findings [17]. Demographic data (age, sex, BMI), procedural characteristics (level, side, and RFA technique), outcome measures (NRS and NDI) [18], patients’ satisfaction, medication use, and complications were reviewed.

### 2.3. Intervention Procedures

We performed a diagnostic procedure on two medial branch levels to block a single facet joint. A diagnostic medial branch block was considered positive when it produced ≥80% short-term pain relief. Only patients meeting this criterion were scheduled for radiofrequency treatment. For each targeted facet joint, at least two corresponding medial branches were blocked. When clinical evaluation suggested multi-level involvement, additional adjacent levels were included. The distribution of treated levels did not differ significantly between the groups. Following this, PRF (at 42 °C for 3 min) or CRF (at 80 °C for 90 s) was offered. The selection of the radiofrequency modality was based on a comprehensive clinical evaluation, including patient-specific anatomical considerations, comorbidities, anticipated risk–benefit profile, and informed patient preference.

**Figure 1:**
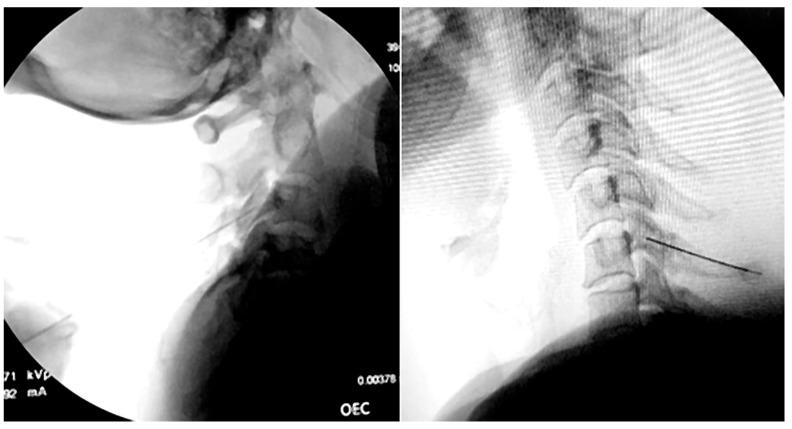
Fluoroscopic images of cervical medial branch radiofrequency procedure.

All procedures have been performed by a single experienced pain physician with over five years of practice. Procedures were performed using a lateral approach in the lateral decubitus position, with the symptomatic side positioned superiorly [19]. Sedation was avoided whenever possible to ensure reliable sensory and motor stimulation responses and was used only in cases of significant patient discomfort. Following sterile preparation and local anesthesia with 1% lidocaine, a 22-gauge RF cannula (10 cm with a 10-mm active tip) (Diros RF Cannula, Diros Technology Inc., Markham, ON, Canada) was advanced to the centre of the articular pillar in the lateral view and the waist in the anteroposterior view, as demonstrated in Figure 1. Particular care was taken to minimize the parallax effect. Appropriate needle positioning was verified using both anteroposterior and lateral fluoroscopic views. After impedance was confirmed under 500 Ohms, sensory testing was carried out at 50 Hz. This should produce paresthesia in the cervical region. Motor stimulation (2 Hz) was performed, with appropriate needle positioning verified by multifidus muscle twitching at <1 V. Contrast (0.1–0.3 mL) was injected at each level to ensure extravascular needle placement and exclude intravascular spread. Next, 1–2 mL of 2% lidocaine was injected to provide analgesia during the RFA. The entire procedure was carried out employing a standard RF lesion generator. Pulsed RF at 42 °C for 180 s was performed after localization. Thermal radiofrequency ablation was applied at a target temperature of 80 °C, maintained for 90 s. The radiofrequency parameters were selected in accordance with previously published protocols and established clinical practice [4,19]. Lesion characteristics, neurophysiological principles, and safety considerations were also considered. For conventional radiofrequency (CRF), a target temperature of 80 °C maintained for 90 s was chosen to achieve an adequate lesion size without unnecessarily increasing the risk of unintended tissue injury and consistent thermocoagulation of the cervical medial branch while limiting excessive lateral heat conduction to adjacent neural and vascular structures. For pulsed radiofrequency (PRF), the temperature was strictly limited to 42 °C for 180 s to avoid structural neural destruction. This temperature threshold is widely recognized as the upper safety limit to prevent irreversible protein denaturation while allowing neuromodulatory electric field effects. The extended duration was selected to enhance cumulative electric field exposure, as PRF efficacy is thought to depend more on electromagnetic field interaction than on thermal mechanisms.

Standardized parameters were applied to all patients in order to reduce operator-dependent variability and improve internal validity, given the known inter-individual anatomical and clinical differences in cervical facet-mediated pain.

Both the PRF and CRF groups received an identical post-lesion injection consisting of 1 mL of 0.125% bupivacaine combined with 2 mg dexamethasone prior to electrode removal. This post-procedural injection protocol was applied uniformly to both groups to minimize potential confounding effects related to corticosteroid administration.

In the absence of serious complications such as hematoma, pneumothorax, or neurological impairments, patients were allowed to leave.

In Figure 1, fluoroscopic images demonstrate a cervical medial branch radiofrequency procedure. Lateral views show needle positioning along the target cervical medial branches under image guidance prior to lesioning.

### 2.4. Measures

Changes in pain were assessed using the Numeric Rating Scale, which varies from 0 to 10. Successful pain reduction was considered to be a ≥50% decrease in the NRS value relative to the pretreatment.

Functional disability was assessed using the NDI, a validated, patient-reported questionnaire consisting of 10 items that evaluate the impact of neck pain on daily activities, with higher total scores indicating greater disability [18].

Analgesic consumption was categorized into four levels, scored between 0 and 3 (0 = no medication, 1 = NSAIDs/non-narcotic medication, 2 = narcotic medications, 3 = routinely narcotic medications).

The patients’ global perceived effect (GPE) was evaluated using a 7-point Likert scale where 1 means ‘much worse’, 2 ‘worse’, 3 ‘slightly worse’, 4 ‘no change’, 5 ‘slightly improved’, 6 ‘good’, and 7 ‘very good’. Scores of 6 (good) and 7 (very good) were predefined as indicating patient satisfaction with the procedure [20].

### 2.5. Data Collection

Outcome measures were recorded prior to the procedure and repeatedly reassessed at 1-, 3-, 6-, and 12-month follow-up intervals.

### 2.6. Sample Size and Statistical Analysis

Statistical analyses were completed utilizing the SPSS software program (version 21.0; IBM Corp., Armonk, NY, USA). Continuous data were presented as mean ± standard deviation or as median (minimum–maximum), depending on the distribution. Categorical variables were reported as frequencies and percentages, and between-group differences were analysed using the chi-square test. Continuous outcomes were compared across groups with the independent-samples *t*-test when data were normally distributed, whereas the Mann–Whitney U test was applied for non-parametric distributions. Repeated measurements were assessed with the Friedman test, and subsequent post hoc analyses were performed for pairwise time-point comparisons. Relationships between variables were explored using Spearman correlation coefficients, with statistical significance defined as *p* < 0.05.

The sample size was calculated using G*Power software (version 3.1). A non-inferiority comparison between PRF and CRF was planned based on the NRS (NRS; 0–10) pain score at 12 months. The non-inferiority margin (Δ) was set at 1.0 point, representing a conservative threshold relative to previously reported minimal clinically important difference (MCID) values for cervical spine pain assessed utilising the Visual Analog Scale (VAS; 0–10) [21,22]. We used a one-sided α = 0.025 and 80% power. For variance planning, published RF data reporting VAS outcomes at 12 months showed group SDs of 1.72 and 1.22, implying a pooled SD ≈ 1.5. Under a two-sample non-inferiority *t*-test with equal allocation, Δ = 1.0 and SD ≈ 1.5 require 35 participants per group; allowing for 10–15% attrition, we planned to recruit 39–42 patients per group (total 78–84). The sample size calculations and the assumptions underlying them are based on data published on the RFA of medial branches [23].

## 3. Results

### 3.1. Patient Flow and Baseline Characteristics

We reviewed the medical notes of 95 patients retrospectively and enrolled 84 patients in the analysis. The average age of the study population was 53.3 years (SD 10.3) (range, 31–70 years).

**Figure 2:**
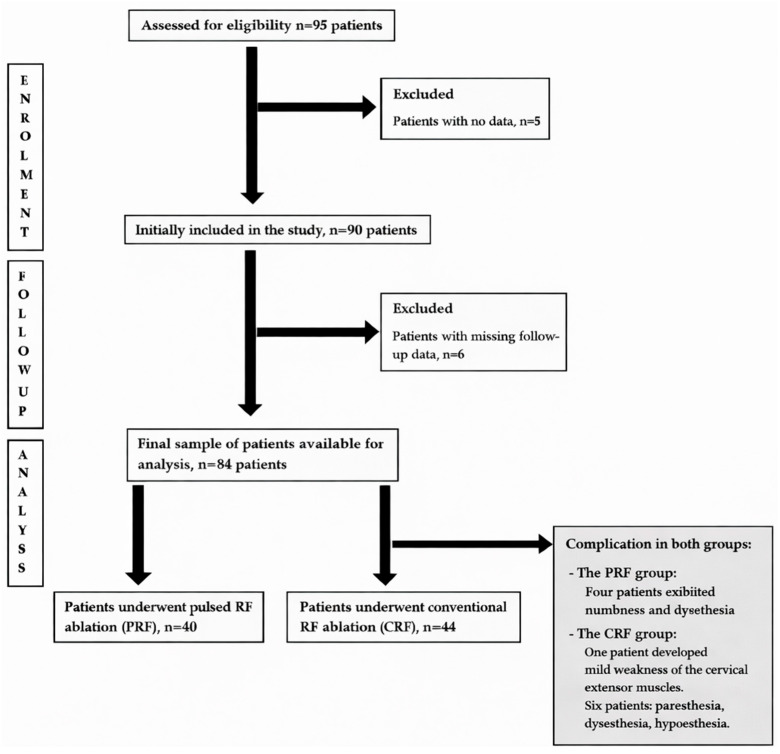
Flow Chart of Case Inclusion and Study Design.

There were nine cases of missing data due to discontinued assessment in the CRF group and two in the PRF group. The subsequent review enrolled 40 individuals in the PRF group and 44 in the CRF group, as shown in Figure 2.

Figure 2 shows a flow diagram of patient selection and the study inclusion process. Of 95 patients assessed for eligibility, 5 were excluded due to lack of baseline data. Ninety patients were initially included; 6 were further excluded because of missing follow-up data. The final analysis included 84 patients, of whom 40 underwent pulsed radiofrequency (PRF) and 44 underwent conventional radiofrequency (CRF) ablation. Procedure-related complications observed in each group are summarized in the diagram.

Baseline patient characteristics are displayed in Table 1. There were no significant differences between the groups in terms of age, sex, smoking status, or initial clinical variables, including NRS and NDI scores, pain duration, distribution of pain site and target levels, and medication use. Baseline pain intensity and disability scores were comparable between the groups.

### 3.2. Treatment Outcomes

The mean values for pain and disability at 1-, 3-, and 6-month follow-ups in both groups were substantially lower than the baseline (*p* < 0.001). In the pulsed RFA group, mean NRS and NDI scores at the 12-month follow-up were lower than baseline values; however, these improvements did not achieve statistical significance (*p* = 0.596 and *p* = 0.548).

Within-group analyses showed that both the PRF and CRF groups experienced improvement in pain intensity and functional disability over time. Baseline and early follow-up (1 and 3 months) comparisons revealed no substantial differences in pain or disability scores in either group. However, at 6- and 12-month evaluations, patients treated with CRF exhibited significantly lower NRS and NDI scores than those treated with PRF (*p* < 0.001), as detailed in Table 2.

For NRS scores, at baseline and 1 month, there was no statistically significant difference between groups (*p* = 0.223 and 0.763), and Bayes Factor analysis indicated that the data were insensitive to detect a difference (BF10 = 1.90 and 1.01), consistent with inconclusive evidence for the null hypothesis. At 3 months, although the Mann–Whitney U test did not reach statistical significance (*p* = 0.072), the Bayes Factor indicated weak evidence for a difference (BF10 = 3.38). At 6 and 12 months, differences were statistically significant (*p* < 0.001) and the Bayes Factor provided extremely strong evidence for a difference (BF10 = 4200 and >100, respectively).

For NDI scores, at baseline, 1 month, and 3 months, there was no statistically significant difference between groups (*p* = 0.160, 0.784, and 0.903), and Bayes Factor analysis indicated that the data were insensitive (BF10 = 1.50, 1.04, and 1.10), consistent with inconclusive evidence for the null hypothesis. At 6 and 12 months, differences were statistically significant (*p* < 0.001), with Bayes Factor providing extremely strong evidence for a difference (BF10 = 865 and >100, respectively).

Post hoc analyses performed following the Friedman test demonstrated that the two treatment groups followed different trajectories over time. Patients treated with PRF experienced a significant reduction in pain and disability scores in the short and intermediate follow-up intervals, in comparison with initial measurements (*p* < 0.001 for pairwise NRS0-1,3,6 and NDI0-1,3,6). This improvement, however, was not sustained, as scores worsened significantly between the 6- and 12-month evaluations (*p* = 0.596 for NRS0-12 and *p* = 0.548 for NDI0-12). In the PRF group, NRS and NDI scores showed significant improvement at 1-, 3-, and 6-month follow-up compared with baseline (all *p* < 0.001). However, no significant difference was observed between baseline and 12-month scores (*p* > 0.05), indicating that the early treatment effect was not sustained at long-term follow-up.

By contrast, patients in the CRF group experienced significant improvement early after treatment, which was maintained or further enhanced at later follow-up assessments (*p* < 0.001 for NRS0-1,3,6,12 and NDI0-1,3,6,12). The presence of significant differences between successive long-term time points indicated a more stable treatment response. These findings suggested that PRF is primarily associated with shorter period clinical improvement, whereas CRF provides more durable analgesic and functional benefits over time. CRF group demonstrated significant and sustained improvements in both NRS and NDI scores at all follow-up time points, including 12 months, compared with baseline (all *p* < 0.001).

These findings suggest that PRF is associated with short- to intermediate-term clinical improvement, whereas CRF provides more durable analgesic and functional benefits.

When a ≥50% reduction in pain intensity from baseline was used to define treatment response, 25 patients (62.5%) in the PRF group met this criterion at 1 month, and 21 patients (52.5%) remained responders at 3 months. The proportion of responders decreased substantially thereafter, with only 2 patients (5.0%) achieving this level of pain improvement at six months and no responders at 12 months. In contrast, the CRF group showed higher and more persistent response rates. A ≥ 50% decrease in pain was noted in 30 individuals (68.2%) at one month, and 33 individuals (75.0%) at three months, with the majority of responders maintaining clinically meaningful pain relief at 6 months [29 patients (65.9%)] and a smaller but still notable proportion at 12 months [7 patients (15.9%)].

At both 1 and 3 months, very good (7), ‘good’ (6), and ‘slightly improved’ (5) scores were similarly high in the PRF and CRF groups, with no meaningful variation in the results of the outcomes (*p* > 0.05). At 6th months, in PRF and CRF groups, satisfaction rates (GPE scores 5–7) were observed in a total of 35 (87.5%) and 43 patients (97.7%) in each group (*p* > 0.05). Accordingly, 17 patients (42.5%) and 39 patients (88.6%) were pleased with the effects after 12 months in both the treatment arms (*p* < 0.001). GPE scores 1–3 were not reported in the study. Patient satisfaction patterns over time are presented in Table 3.

Medication use was classified as no medication (0), NSAIDs/non-narcotic drug (1), narcotic (2), and routine narcotic use (3). At baseline, medication use patterns were similar in the PRF and CRF treatment arms, with no noticeable change in groups (*p* > 0.05). Medication requirements decreased significantly in both PRF and CRF populations over time (*p* < 0.001), reflecting a progressive shift from opioid-dominant baseline use toward nonsteroid drug use or complete discontinuation. At the 1- and 3-month assessments, medication requirements remained comparable in the two groups (*p* > 0.05). However, by the 6-month assessment, a notable difference had emerged (*p* < 0.001), with a relatively high percentage of individuals in the CRF group managed without medication or with non-narcotic drugs (*p* < 0.05). This difference became more pronounced at the 12-month assessment, where narcotic use was lower in the patients who received CRF than in the PRF patients (*p* < 0.001).

Correlation analyses revealed no clinically meaningful associations between demographic variables (sex, BMI, smoking status), pain duration, treatment side, or procedural level and treatment outcomes (NRS, NDI, or GPE) in either the PRF or CRF groups (all *p* > 0.05). Age demonstrated only weak correlations with selected follow-up NRS measures at 3, 6, and 12 months. In contrast, baseline NRS and NDI scores showed modest-to-strong correlations with several subsequent clinical outcomes (*p* < 0.05).

There were no significant unfavorable complications linked with the intervention that were observed following the PRF or CRF procedure (*p* = 0.526). In the PRF group, four patients experienced mild transient numbness and dysesthesia in the posterior cervical region. These symptoms were self-limited, required no additional treatment, and resolved completely within several days. In the CRF group, one patient developed mild cervical muscle weakness following RFA of the C2–C6 medial branches. Neurological examination revealed no motor deficit involving the upper extremities. The weakness was limited to cervical extension, did not interfere with daily activities, and resolved spontaneously within two weeks without specific treatment. Additionally, six patients in the CRF group reported transient sensory disturbances (paresthesia, dysesthesia, or hypoesthesia) in the posterior cervical region, all of which were mild in severity and resolved completely within one week. No permanent neurological deficits, vertebral artery injury, nerve root injury, infection, or hematoma were observed.

## 4. Discussion

The present study demonstrated that both PRF and CRF lesioning of the CMBs resulted in substantial relief in pain intensity and functional disability at all follow-up points except 12th months. Only the cases in the CRF group continued to show a substantial improvement compared with initial assessments at the 12-month point. At the 1- and 3-month evaluations, both groups demonstrated comparable improvements in pain, disability, medication use, and patient satisfaction, with no notable differences among the treatments (*p* > 0.05). In contrast, CRF was associated with significantly better clinical results at 6 and 12 months without increasing the rate of complications (*p* < 0.001). PRF may be associated with a shorter duration of clinical benefit, thereby necessitating more frequent repeat procedures to maintain symptom control.

No studies have directly compared the cervical facet medial branch RFA procedures for neck pain. The largest number of reports have investigated lumbar medial branch radiofrequency ablation techniques for lower back pain. Li et al. concluded that no statistically substantial differences were observed in pain, functional impairment, and life quality scores between the pulse and traditional RF groups at the 3-month point in cases of lumbar pain [23]. In their review, Qi et al. investigated the effectiveness of various radiofrequency techniques in relieving lumbar facet-related pain. The efficacy of RF technologies [pulse, traditional radiofrequency, low-temperature plasma RFA (LTPRFA), and cooled] was reported to be, in the short term, best for LTPRFA, with pulse, traditional, and conventional RF showing the subsequent highest efficiency with a non-significant difference. The order of efficacy was LTPRFA, followed by cooled and traditional, which were found to be equally efficacious, and pulse RF for medium term, while in the long term, LTPRFA, cooled and traditional radiofrequency were found to be beneficial and no meaningful difference; and PRF was found to be poorly efficacious [24]. In another study, over a more extended timeframe of up to two years by Yılmaz et al., which compared the efficacy of conventional RF and crioablation, concluded that CRF demonstrated better functionality, and lower pain levels were observed at twelve and eighteen months, while by twenty-four months, both radiofrequency ablation and cryoablation demonstrated weaker but similar impacts (*p* > 0.05) [25]. Evidence from studies on back pain indicated that CRF could provide clinical benefits lasting longer than one year, whereas PRF provided more short- and medium-term relief. Our findings in the cervical region were consistent with these results and supported the greater durability of CRF over longer follow-up periods.

Two randomized controlled trials have assessed radiofrequency administered to the cervical dorsal root ganglion (DRG) [26,27]. In one trial, Van Kleef et al. demonstrated significant short-term efficacy at 2 months, with a favorable number needed to treat (NNT) of 1.4 [26]. However, Slappendel et al. reported no meaningful difference between low-temperature (40 °C) and conventional high-temperature (67 °C) radiofrequency treatment, despite both protocols producing a notable decrease in VAS values at 1.5 and 3 months [27].

PRF stimulation is a minimally neuro-destructive intervention that is used in clinical practice settings for managing pain associated with the facet joint, with a reported low incidence of significant complications [28]. The primary benefits of PRF therapy are that it is a relatively comfortable therapeutic modality that does not cause heat-related tissue injury. PRF generates an electrical environment that produces a targeted or widespread impact on immune responses, thereby hindering the development of long-term pain [29]. The presence of noxious signals throughout the pain signalling system may be reduced, and the electromagnetic area generated by PRF has the potential to modify signal transmission [8]. Moreover, PRF therapy has been shown to reduce microglial reactivity in the dorsal horn of the spinal cord [29]. Because microglia release various chemicals, including pro-inflammatory mediators, which are related to the occurrence of prolonged pain, the attenuation of microglia activity has the capacity to modify the experience of pain [29]. In addition, the application of PRF stimulation has been demonstrated to induce microscopic damage to unmyelinated C fibres, which are vital for transmitting signals of pain [30]. The point at which neural cells begin to be damaged is 45 °C, but pRFA ensures that this temperature is not achieved [31]. Chang et al. reported in their study that PRF therapy of the CMBs may efficiently control chronic facetojenic pain, and half of the subjects experienced effective pain alleviation (a decrease in pain of at least 50%) and positive feedback; this beneficial effect persisted for at least three months [15]. Recent experimental findings have indicated that the electrical area may contribute to the enhanced expression of immediate-early genes (IEG), particularly c-fos [32]. One hypothesis suggested that c-fos proteins, which are generated by IEG expression, alter neuronal signaling. In line with the proposed neuromodulatory mechanisms, our findings suggested that PRF is clinically useful in treating cervical facet-mediated pain, particularly during the early post-treatment phase. However, the shorter duration of improvement experienced by the PRF versus the CRF might reflect the non-destructive nature of PRF, suggesting that its effects are predominantly transient and modulatory rather than long-lasting neuroablative.

Liliang et al. stated that PRF stimulation of the cervical medial branches alleviated pain caused by zygapophysial joint dysfunction following whiplash trauma, in addition to a reduction in medication intake. Eleven (78.3%) patients continued to experience substantial pain improvement at the six-month follow-up, and nine (64.3%) patients reported significant pain relief after one year. At the 1-year assessment, 10 individuals (71.4%) had experienced a reduction in their medication consumption. None of the cases has documented complications or discomfort. They suggested that pulse RF is a safe, efficient, and sustainable pain relief strategy that reduces medication requirement [33].

Conventional radiofrequency (CRF) involves thermal coagulation of the targeted medial branches, attained by positioning the radiofrequency needle alongside the nerve. The heat generated at the probe tip creates a controlled lesion, leading to focal nerve disruption and thereby reducing nociceptive signal transmission along the sensory pathway [34]. In this respect, the CRF of MBBs is a specialised procedure. Thermal equilibrium is attained once the needle tip has achieved the required temperature, and hence, optimal lesion size is obtained after 60–90 s. CRF does not definitively damage its goal nerves; it only causes coagulation of their distal fibres. The DRG maintains its functionality, and the nerves gradually regrow following coagulation, a process that takes a few months. Once the nerves have regenerated, the pain may reappear. Therefore, CRF should not be considered a long-term treatment or definitive cure for neck pain, similar to PRF. On the other hand, the therapeutic response to cervical RFA is variable; while many patients benefit from the intervention, a subset may fail to respond or may even report an aggravation of pain. It has been suggested that in patients who obtain ≥30% pain reduction lasting at least three months after the initial intervention, repeat RFA can be considered. However, it is generally recommended that the procedure should not be performed more than twice within a year [7]. However, combined evidence from RCTs and observational studies suggests that cervical radiofrequency neurotomy can provide pain relief lasting six months or longer, with Level II strength of evidence, supporting its role as a durable interventional option for chronic neck pain [35]. Consistent with these observations, our findings also showed that CRF led to significant and lasting improvements in pain and disability outcomes during the follow-up period. Notably, the superior durability observed in the CRF treatment group at six and twelve months further supports its role as a longer-lasting intervention for cervical facet-mediated pain compared with PRF.

The diagnostic confirmation of facet-mediated pain is commonly achieved using MBBs, which are expected to produce short-term pain relief following the administration of local anaesthetics. The predictive value of these blocks for clinical outcomes after CMB radiofrequency ablation has been well established, with significant improvements reported in both pain intensity and functional status [36]. However, the degree of pain reduction following diagnostic blockades does not appear to influence the subsequent outcome of radiofrequency treatment. Cohen et al. reported that no clinically significant difference in radiofrequency treatment responses was observed among individuals who achieved 50–79% and ≥80% pain relief, respectively, after diagnostic blocks [37]. Furthermore, Taylor et al. showed no substantial differences in clinical outcomes after CMB radiofrequency ablation between patients selected using strict dual-block criteria (100% pain improvement) and individuals selected using relatively relaxed criteria (80–99% pain improvement), suggesting that overly restrictive selection protocols may unnecessarily exclude patients from receiving an effective intervention [3]. On the other hand, Cohen et al. investigated the impact of diagnostic block protocols and reported that denervation success was 39% with a single block and improved to 64% when dual blocks were performed [38]. Importantly, the diagnostic MBBs are associated with a notable false-positive rate of 20–40% [24]. Potential explanations for the false-positive rate include a diagnostic placebo response, the confounding impact of sedation, and the possibility of local anaesthetic affecting nociceptors outside the primary injection site. In the present study, a diagnostic block was deemed successful if it provided 80% or greater pain relief. For diagnostic confirmation, a single MBB was applied across at least two suspected facet levels to confirm the facet-mediated origin of pain, to reduce patient burden and procedural risk, while maintaining clinical feasibility and applicability in ordinary conditions.

Beyond the procedure itself, various additional circumstances may influence the effectivity of RF treatment. Age has been suggested as one such factor, with some studies reporting slightly better outcomes in older patients [39]. Candan et al. observed slightly greater pain reduction in patients aged 50 years or older, with NRS improvements of 63.4%, compared to 58.4% in younger patients. Their findings suggested that advanced age may be associated with better RF responses in lumbar pain [39]. In our analysis, age demonstrated only weak correlations with selected follow-up NRS measures. Although baseline NRS and disability scores showed modest correlations with follow-up measures, no demographic or procedural characteristic strongly predicted treatment success independently. The use of analgesics before RF treatment was found to diminish the effectiveness of RF therapy [24]. It was also proposed that paraspinal sensitivity was the main clinical factor influencing treatment success [37]. Pronounced paravertebral tenderness on palpation may reflect local soft-tissue reactions secondary to an underlying facet joint injury; however, this finding is not diagnostically specific. In the cervical spine, painful zygapophysial joints and myofascial trigger points produce overlapping clinical features, and no diagnostic method based on physical examination has been validated to reliably differentiate between these conditions [40]. This underscores the continued importance of diagnostic blocks and careful patient selection to optimize clinical response.

In the research conducted by Khan et al., it was recommended that MBBs under CT fluoroscopy-guidedprovide anatomic visualization through both bone and soft tissue windows [1]. They suggested that CT-guided CMB denervation is a viable option when compared with traditional fluoroscopic techniques, with the added benefits of accurate needle positioning and the ability to visualize neighboring soft-tissue structures that may be at risk despite not being the primary target. Nevertheless, all the procedures in the current analysis were carried out under fluoroscopy guidance in accordance with established procedural standards, which remains a widely accepted, practical, and effective imaging modality for CMB interventions.

Potential complications following radiofrequency treatment encompass ataxia, short-term pain exacerbation, sensory symptoms, impaired sensation in the skin, and dizziness, which have been reported in 19% to 97% of cases [4]. The broad spectrum of incidence rates may be explained by variations in the cervical regions addressed or RF procedures employed. A review of radiofrequency denervation procedures revealed a very low incidence of minor adverse events (1%) and no new motor or sensory impairment. With appropriate patient selection, RF facet denervation appears to be a highly effective intervention, with reported NNT values between 1.1 and 1.5 [11]. Nevertheless, a study indicated that RFA of the upper cervical level is related to a higher incidence of neuritis [41]. Consequently, PRF has been introduced to prevent persistent or severe thermal reactions related to the CRF procedure. Malaithong et al. concluded that the combination of conventional and pulsed RF groups (n = 17) demonstrated a significantly reduced incidence of complications, including dysesthesic sensations, numbness, and hypersensitivity reactions, when compared to the conventional RF group (n = 22) [4]. It was proposed that the combination of CRF and PRF could be utilized for the therapeutic approach to cervical facet joint-related pain, exhibiting successful outcomes and sustained length of symptom control that is comparable to that of CRF alone, while concurrently demonstrating a notable reduction in postoperative complications. In the current study, both PRF and CRF were generally well tolerated, and no serious neurological complications were encountered. Minor side effects were transient and resolved without additional treatment. Moreover, complication rates were comparable between the two techniques, supporting the overall safety of cervical medial branch RF when performed with appropriate patient selection and procedural care.

This research had certain limitations. One of the drawbacks of this work was that it was conducted retrospectively. The unavailability of a placebo-controlled control group was another limitation. The trial did not involve a dual block in the assessment phase. Nonetheless, updated guidelines have suggested that performing a single diagnostic block prior to cervical RFA is generally adequate, since a double block might result in a substantial number of false negative responses [42]. An additional limitation was the absence of more prolonged long-term follow-up data. Despite these limitations, the study also had considerable plus points. To date, this appears to be the first study to directly compare pulsed and conventional radiofrequency techniques for the treatment of cervical facet-related pain. Furthermore, treatment efficacy was assessed using validated and objective clinical outcome instruments, including the NRS, NDI, and GPE, allowing a reliable evaluation of pain intensity, functional improvement, and patients’ satisfaction.

## 5. Conclusions

Both pulsed and conventional radiofrequency denervation of the cervical facet joint medial branch supply provided significant pain relief, functional improvement, and reduced medication requirements in patients with persistent neck pain at 1st, 3rd, and 6th months. Conventional radiofrequency demonstrated superiority in terms of clinical benefit at 6 months, and sustained improvements were observed at 12 months only in patients treated with CRF. Overall, CRF appears to offer a longer-lasting therapeutic advantage in controlling symptoms over time. If carried out by trained clinicians, both conventional and pulsed radiofrequency interventions appear to be safe and effective treatment options. Nevertheless, large-scale, randomized, controlled, prospective investigations are warranted to better understand the contribution of pulsed and conventional radiofrequency in the therapeutic management of cervical zygapophysial joint-mediated pain.

## Figures and Tables

**Table 1:** Demographic and Clinical Features of Subjects.

	PRF Group (n = 40)	CRF Group (n = 44)	*p*-Value
Age (years), Mean ± SD	54.2 ± 9.3	52.6 ± 11.2	0.472 *
Sex, n (%)			0.851 **
Male	21 (52.5)	24 (54.5)	
Female	19 (47.5)	20 (45.5)	
BMI (kg/m^2^), Mean ± SD	24.6 ± 2.8	23.7 ± 2.6	0.137 *
Pain duration (years), n (%)			0.993 **
<1 year	15 (37.5)	16 (36.4)	
1–5 years	15 (37.5)	17 (38.6)	
>5 years	10 (25.0)	11 (25.0)	
Smoking, n (%)			0.231 **
Yes	18 (45.0)	17 (38.6)	
No	22 (55.0)	27 (61.4)	
Site, n (%)			0.061 **
Left	20 (50.0)	21 (47.7)	
Right	20 (50.0)	23 (52.3)	
Target level, n (%)			
C2–4	3 (7.5)	5 (11.4)	0.715 ^¥^
C2–5	4 (10.0)	6 (13.6)	0.741 ^¥^
C2–6	4 (10.0)	6 (13.6)	0.741 ^¥^
C2–7	3 (7.5)	4 (9.1)	0.999 ^¥^
C3–5	4 (10.0)	5 (11.4)	0.999 ^¥^
C3–6	6 (15.0)	3 (6.8)	0.298 ^¥^
C3–7	3 (7.5)	3 (6.8)	0.999 ^¥^
C4–6	5 (12.5)	3 (6.8)	0.469 ^¥^
C4–7	4 (10.0)	4 (9.1)	0.999 ^¥^
C5–7	4 (10.0)	5 (11.4)	0.999 ^¥^
Baseline medication use, n (%)			0.562 ^¥^
No medication (0)	2 (5.0)	3 (6.8)	
Non-narcotic medication (1)	7 (15.0)	13 (25.0)	
Narcotic medication (2)	18 (42.5)	18 (45.5)	
Routinely narcotic medication (3)	13 (37.5)	10 (22.7)	
Baseline NRS, Mean ± SD	7.33 ± 1.31	7.64 ± 1.22	0.223 ***
Baseline NDI, Mean ± SD	23.9 ± 5.5	24.9 ± 5.4	0.160 ***

BMI: Body Mass Index; NRS: Numeric Rating Scale; NDI: Neck Disability Index. Data are presented as mean ± standard deviation or number (%). * Independent Samples *t*-test, ** Chi-square test, ^¥^ Fisher’s Exact Test, *** Mann–Whitney U test.

**Table 2:** Evaluation of NRS and NDI scores at baseline and 1, 3, 6, and 12 months.

Outcome	Time Point	PRF Group (Median, Min–Max)	CRF Group (Median, Min–Max)	*p*-Value ^b^	BF10	Interpretation
NRS	Baseline	7 (5–10)	8 (5–10)	0.223	1.90	Data insensitive
	1 month	4 (2–5) *	3 (2–6) *	0.763	1.01	Data insensitive
	3 months	4 (2–6) *	3 (3–6) *	0.072	3.38	Weak evidence for difference
	6 months	5 (3–7) *	3 (2–6) *	<0.001	4200	Extremely strong evidence for difference
	12 months	7 (5–9)	5 (3–7) *	<0.001	Very High	Extremely strong evidence for difference
	*p*-value ^a^	<0.001	<0.001			
NDI	Baseline	24 (14–37)	25 (10–35)	0.160	1.50	Data insensitive
	1 month	13 (4–25) *	13 (5–24) *	0.784 ^c^	1.04	Data insensitive
	3 months	13 (4–27) *	14 (5–22) *	0.903	1.10	Data insensitive
	6 months	18 (6–27) *	14 (5–23) *	<0.001 ^c^	865	Extremely strong evidence for difference
	12 months	22 (14–37)	16 (8–23) *	<0.001 ^c^	Very High	Extremely strong evidence for difference
	*p*-value ^a^	<0.001	<0.001			

NRS: Numeric Rating Scale; NDI: Neck Disability Index; m: month; ^a^ Friedman test (within-group comparisons); ^b^ Mann–Whitney U test (between-group comparisons); ^c^ Independent Samples Test; * indicates measurement intervals that differ significantly from baseline values (*p* < 0.05). BF10: Bayes Factor for alternative hypothesis over null hypothesis.

**Table 3:** Distribution of Global Perceived Effect (GPE) Scores During Follow-Up.

Time Point	Group	No Change	Slightly Improved	Good	Very Good	*p*-Value *
GPE at 1 m	pulse	0 (0.0%)	11 (27.5%)	18 (45.0%)	11 (27.5%)	0.769
	conventional	2 (4.5%)	13 (29.5%)	18 (40.9%)	11 (25.0%)	
GPE at 3 m	pulse	1 (2.5%)	18 (45.0%)	15 (37.5%)	6 (15.0%)	0.487
	conventional	3 (6.8%)	15 (34.1%)	15 (34.1%)	11 (25.0%)	
GPE at 6 m	pulse	5 (12.5%)	25 (62.5%)	10 (25.0%)	0 (0.0%)	0.020
	conventional	1 (2.3%)	23 (52.3%)	14 (31.8%)	6 (13.6%)	
GPE at 12 m	pulse	23 (57.5%)	17 (42.5%)	0 (0.0%)	0 (0.0%)	<0.001
	conventional	5 (11.4%)	22 (50.0%)	11 (25.0%)	6 (13.6%)	

GPE: Global Perceived Effect, m: month, * Fisher’s Exact Test.

## Data Availability

The datasets generated and analyzed during the current study are available from the corresponding author on reasonable request. The data are not publicly available due to ethical restrictions and institutional policies.

## Abbreviations

The following abbreviations are used in this manuscript:

CNP	Chronic Neck Pain
NRS	Numerical Rating Scale
RFA	Radiofrequency Ablation
PRFA	Pulsed Radiofrequency Ablation
CRFA	Conventional Radiofrequency Ablation
CMBs	Cervical Medial Branches
NDI	Neck Disability Index
BMI	Body Mass Index
PRF	Pulsed Radiofrequency
CRF	Conventional Radiofrequency
GPE	Global Perceived Effect
MBBs	Medial Branch Blocks
MCID	Minimal Clinically İmportant Difference
VAS	Visual Analog Scale
DRG	Dorsal Root Ganglion
IEG	Immediate Early Genes
